# Alzheimer’s Disease: Causal Effect between Obesity and APOE Gene Polymorphisms

**DOI:** 10.3390/ijms241713531

**Published:** 2023-08-31

**Authors:** Tianyu Zhao, Tangsheng Zhong, Meishuang Zhang, Yang Xu, Ming Zhang, Li Chen

**Affiliations:** 1 Department of Pharmacology, College of Basic Medical Sciences, Jilin University, 126 Xinmin Street, Changchun 130012, China; zhaoty22@mails.jlu.edu.cn (T.Z.); yxu22@mails.jlu.edu.cn (Y.X.); 2 School of Nursing, Jilin University, 965 Xinjiang Street, Changchun 130012, China; zhongtangsheng@jlu.edu.cn (T.Z.); zhangmeishuang@jlu.edu.cn (M.Z.)

**Keywords:** obesity, Alzheimer’s disease, APOE gene polymorphisms, Mendelian randomization, causal association

## Abstract

Currently studies on the correlation between obesity and Alzheimer’s disease (AD) are still unclear. In addition, few indicators have been used to evaluate obesity, which has failed to comprehen-sively study the correlations between body fat mass, body fat distribution, and AD. Thus, this study innovatively utilized bioinformatics and Mendelian randomization (MR) to explore the key targets of obesity-induced AD, and investigate the causal associations between different types of obesity and key targets. The common targets of obesity and AD were screened using the GeneCards database, and functional and pathway annotations were carried out, thereby revealing the key target. MR analysis was conducted between body anthropometric indexes of obesity and the key target using an IVW model. Bioinformatics analysis revealed Apolipoprotein E (APOE) as the key target of obesity-induced AD. MR results showed that body mass index (BMI) had a negative causal association with APOE2, while body fat percentage (BFP) and trunk fat percentage (TFP) had no significant causal association with APOE2; BMI, BFP, and TFP had a negative causal association with APOE3, and none had any significant causal association with APOE4. In conclusion, there is a correlation between obesity and AD, which is mainly due to the polymorphism of the APOE gene rather than adipose tissue distribution. APOE3 carriers may be more susceptible to obesity, while the risk of AD caused by APOE2 and APOE4 may not be induced by obesity. This study sheds new light on current disputes. At the same time, it is suggested to regulate the body fat mass of APOE3 carriers in the early stage, and to reduce the risk of AD.

## 1. Introduction

With the improvement in people’s living status and the change in dietary structure, obesity has become a serious worldwide public health problem, threatening human health and life. In 2013, the American Medical Association officially defined obesity as a disease. Obesity affects almost all ages and has become a major worldwide medical and public health problem. Alzheimer’s disease (AD) is a common neurodegenerative disease in the elderly, and the most common cause of dementia. Obesity is a risk factor for many cardiovascular and cerebrovascular diseases, and its relationship with AD has become a hot research topic in nutrition science and neuroscience in the recent years. The 2022 Alzheimer’s Disease Facts and Figures published by the Alzheimer’s Association highlights that obesity is an important risk factor for AD [1]. At present, research on obesity and AD has made great progress to a certain extent. However, there are still many disputes and controversies regarding whether obesity is more likely to cause AD, and the influence of different types of obesity on AD, with inconsistency in research conclusions and mechanisms.

Some researchers have found that the increased incidence of obesity is associated with a greater risk of developing AD [2,3]. A meta-analysis by Profenno et al. [4] showed that obesity was a risk factor for AD (OR: 1.59, 95% CI 1.02–2.5). In a long-term observational study of 10,276 members of a Kaiser Permanente health care program in Northern California, Whitmer et al. [5] found that 12.2% of the 713 patients diagnosed with AD were obese (compared with 9.9% in the normal population). However, other researchers, such as Beydoun et al. [6], conducted a meta-analysis of ten cohort studies including people aged 40 to 80 years from 1995 to 2007, and found that there was a U-shaped relationship between obesity and AD, and both obesity and low BMI could increase the risk of AD. Meanwhile, several cross-sectional studies have shown that people with dementia have lower levels of BMI compared with those without in the elderly population [7,8]. In addition, the study of Nourhashémi et al. [9] found that there was no significant relationship between BMI and AD after adjusting for other covariates.

According to the World Health Organization (WHO), obesity is a condition in which the body converts excess energy into excess fat, which accumulates in the body. However, with the same amount of fat, different people have very different risks of developing AD, and studies have shown that these differences are due to different fat distribution phenotypes. Based on where the fat is distributed in the body, obesity is mainly divided into systemic obesity (or peripheral obesity) and central obesity (or visceral obesity/abdominal obesity). Systemic obesity refers to the accumulation of fat in the extremities and under the skin, with more fat in the lower body, also known as “pear-shaped obesity”. Central obesity mainly has the fat gathering in the trunk and inside the abdomen, with increased visceral fat, thickened waist and relatively thin limbs, which is often called “apple-shaped obesity” [10]. Compared with systemic obesity, central obesity is often accompanied by reduced hippocampal volume and white matter ischemia, which are the basis of cognitive decline in dementia and are risk factors for cognitive impairment and AD [11,12]. A systematic review and meta-analysis by Tang et al. [13], involving more than 5 million people in 21 studies, found that central obesity was associated with an increased risk of cognitive impairment and dementia, particularly in people over 65 years of age, with a 13% increased risk. Long-term follow-up studies have shown an increased risk of AD later in life in individuals with higher BMIs in their 30 s to 50 s, especially in those with central obesity [14,15]. In addition, a dose–response meta-analysis confirmed that central obesity was a risk factor for cognitive impairment and dementia [16].

Different types of obesity are evaluated with different body measures. BMI, which reflects the total body fat mass, is currently the most common and simplest measure of obesity, and the best indicator to assess systemic obesity. According to WHO Technical Report Series (2000) [17], the normal range of adult BMI was defined as 18.5–24.9 kg/m^2^, BMI ≥ 25 kg/m^2^ for overweight, and BMI ≥ 30 kg/m^2^ for obesity. These BMI values are age-independent and the same for both sexes. However, BMI cannot accurately measure the distribution of fat and the percentage of fat mass to body weight, and its ability to distinguish between fat mass and fat-free mass is weak; thus, BMI is not a good indicator for central obesity. Studies have reported that higher trunk fat mass may be associated with a greater risk of visceral fat accumulation, showing an association with human longevity; as a marker of central obesity, it is significantly associated with low cognitive levels and has higher sensitivity and accuracy than BMI [18].

In view of the controversial conclusions on the correlation between obesity and AD and the relatively simple indicators for evaluating obesity in previous relevant studies, which have not comprehensively investigated the correlations between body fat mass, body fat distribution, and AD, this study innovatively used bioinformatics to explore the common targets of obesity and AD, and built a “target-function” network of obesity-induced AD. In addition, large-scale GWAS data were analyzed using Mendelian randomization approach, revealing the causal associations between different types of body anthropometric indexes of obesity and related targets, which brought new knowledge regarding whether obesity was more likely to cause AD and the influence of different fat distribution on AD.

## 2. Results

### 2.1. Bioinformatics Analysis

#### 2.1.1. Obesity- and AD-Related Targets, and PPI Network of Overlapping Targets

**Figure 1:**
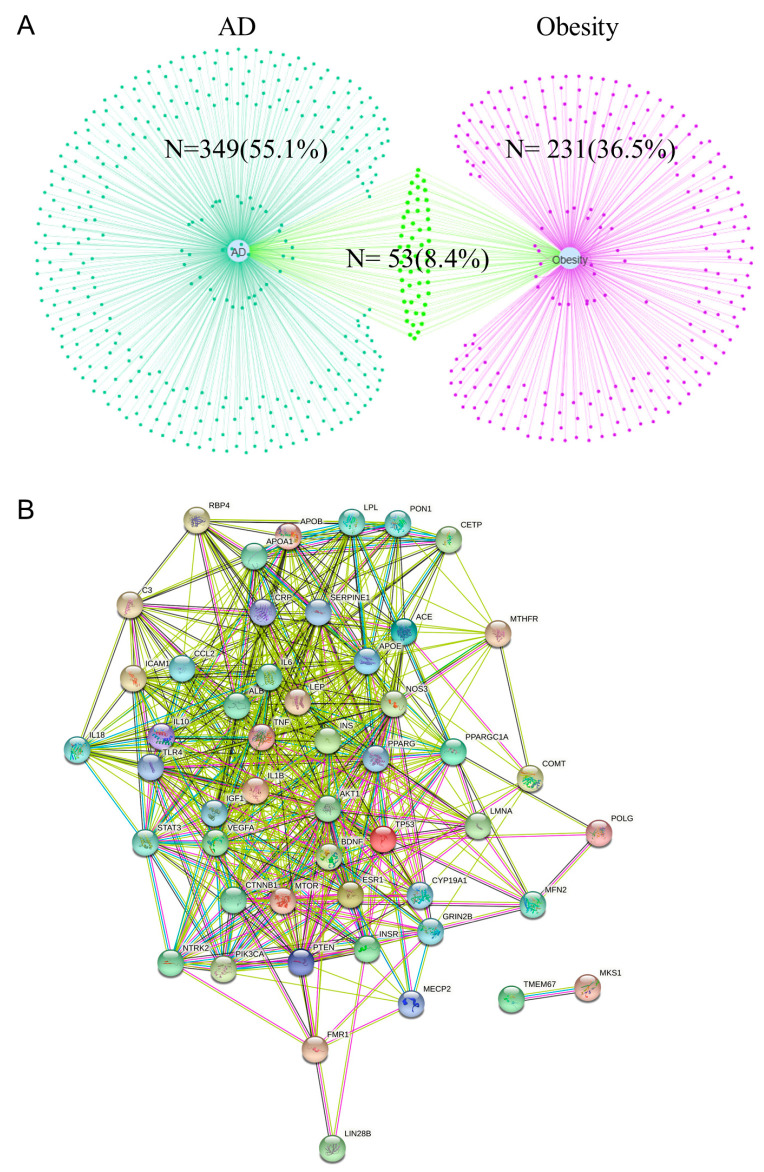
Overlapping targets between obesity and AD ((**A**) Venn diagram of obesity and AD mapping targets. cyan dots on the left are AD-specific targets, green dots in the middle are overlapping targets between obesity and AD, purple dots on the right are obesity-specific targets; (**B**) Protein-protein interaction of 53 overlapping targets. the network nodes are proteins, the edges represent the predicted functional associations).

A total of 284 obesity-related and 402 AD-related targets were screened from the GeneCards database. Further, the two sets were mapped to each other using the EVenn visualization mapping website to obtain 53 overlapping targets between obesity and AD (Figure 1A). The PPI network of the 53 overlapping targets was visualized using the STRING database (Figure 1B).

#### 2.1.2. Functional and Pathway Annotations of Overlapping Targets between Obesity and AD

##### Biological Processes to GO Enrichment Analysis Results of Overlapping Targets between Obesity and AD

**Figure 2:**
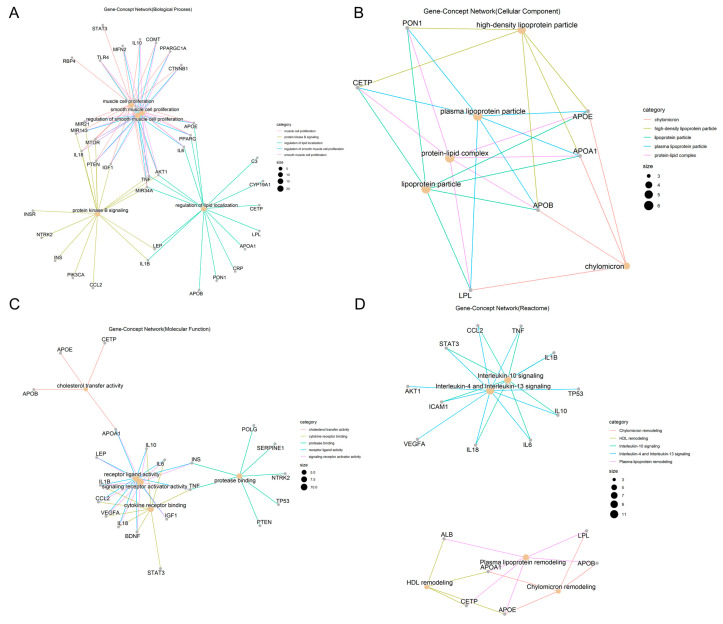
Top five functional and pathway annotations of overlapping targets between obesity and AD ((**A**) biological processes; (**B**) cellular components; (**C**) molecular functions; (**D**) reactome pathways). khaki dots represent each biological process, cellular component, molecular function, reactome pathway, respectively; the size of the khaki dots was positively correlated with the number of targets included. grey dots represent targets.

The GO enrichment results identified 2821 biological processes in which overlapping targets of obesity and AD were involved (*p.adjust* ≤ 0.05), and the top five most significant biological processes selected for Gene-Concept network construction based on 7.30 × 10^−21^ ≤ *p.adjust* ≤ 3.27 × 10^−16^ (Figure 2A) were as follows: ① muscle cell proliferation, in which 20 targets were involved, including APOE, TNF, and IL18; ② the regulation of smooth muscle cell proliferation, in which 18 targets were involved, including APOE, TNF, and IL18; ③ smooth muscle cell proliferation, in which 18 targets were involved, including APOE, TNF, and IL18; ④ the regulation of lipid localization, in which 16 targets were involved, including APOE, TNF, and APOB; ⑤ protein kinase B signaling, in which 16 targets were involved, including TNF, IL18, and IL1B.

##### Cellular Components to GO Enrichment Analysis Results of Overlapping Targets between Obesity and AD

The GO enrichment results identified 77 cellular components in which overlapping targets of obesity and AD were involved (*p.adjust* ≤ 0.05), and the top five most significant cellular components selected for the gene–concept network construction based on 5.42 × 10^−10^ ≤ *p.adjust* ≤ 3.38 × 10^−8^ (Figure 2B) were as follows. ① Plasma lipoprotein particles: six targets were in this cellular component, including APOE, APOA1, PON1, APOB, LPL, and CETP. ② Lipoprotein particles: six targets were in this cellular component, including APOE, APOA1, PON1, APOB, LPL, and CETP. ③ Protein–lipid complex: six targets were in this cellular component, including APOE, APOA1, PON1, APOB, LPL, and CETP. ④ High-density lipoprotein particle: five targets were in this cellular component, including APOE, APOA1, PON1, APOB, and CETP. ⑤ Finally, chylomicron: four targets were in this cellular component, including APOE, APOA1, APOB, and LPL.

##### Molecular Functions to GO Enrichment Analysis Results of Overlapping Targets between Obesity and AD

The GO enrichment results identified 125 molecular functions in which overlapping targets of obesity and AD were involved (*p.adjust* ≤ 0.05), and the top five most significant molecular functions selected for the gene–concept network construction based on 2.66 × 10^−6^ ≤ *p.adjust* ≤ 3.68 × 10^−5^ (Figure 2C) were as follows. ① Receptor ligand activity: 12 targets have this molecular function, including APOA1, TNF, and IL18. ② Signaling receptor activator activity: 12 targets have this molecular function, including APOA1, TNF, and IL18, ③ Cytokine receptor binding: nine targets have this molecular function, including TNF, IL18, and IL6. ④ Protease binding: seven targets have this molecular function, including TNF, PTEN, and INS. ⑤ Lastly, cholesterol transfer activity: four targets have this molecular function, including APOE, APOA1, APOB, and CETP.

##### Reactome Pathways of Overlapping Targets between Obesity and AD

Reactome pathway results identified 101 pathways in which overlapping targets of obesity and AD were involved (*p.adjust* ≤ 0.05), and the top five most significant reactome pathways selected for the gene–concept network construction based on 4.59 × 10^−10^ ≤ *p.adjust* ≤ 6.86 × 10^−6^ (Figure 2D) were as follows. ① Interleukin-4 and Interleukin-13 signaling: 11 targets are involved in this pathway, including TNF, IL18, and IL6. ② Interleukin-10 signaling: eight targets are involved in this pathway, including TNF, IL18, and IL6. ③ Plasma lipoprotein remodeling: six targets are involved in this pathway, including APOE, APOA1, ALB, APOB, LPL, and CETP. ④ Chylomicron remodeling: four targets are involved in this pathway, including APOE, APOA1, APOB, and LPL. ⑤ HDL remodeling: four targets are involved in this pathway, including APOE, APOA1, ALB, and CETP.

##### Frequency of Involvement of Overlapping Targets between Obesity and AD in Top Five Functional and Pathway Annotations

**Figure 3:**
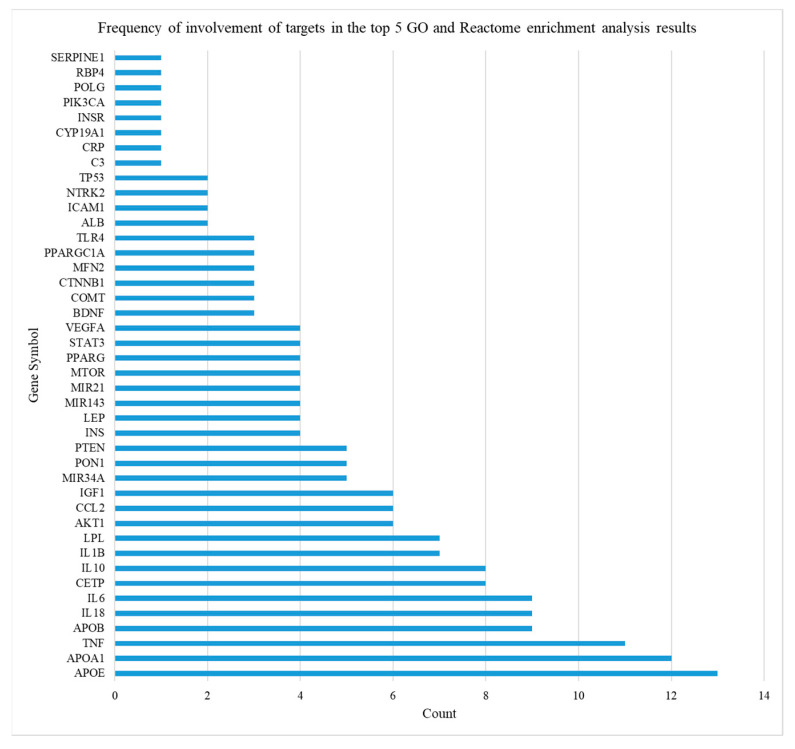
Frequency of involvement of targets in the top five GO and Reactome enrichment analysis results.

The frequencies of relevant targets in the top five biological processes, cellular components, molecular functions, and reactome pathway analyzed in the above sections were determined. A total of 42 targets were involved in the top five functional and pathway annotations and were sorted from the highest to lowest frequency. Three targets had a frequency > 10, of which the one with the highest frequency was APOE (Figure 3).

### 2.2. Mendelian Randomization Analysis

**Figure 4:**
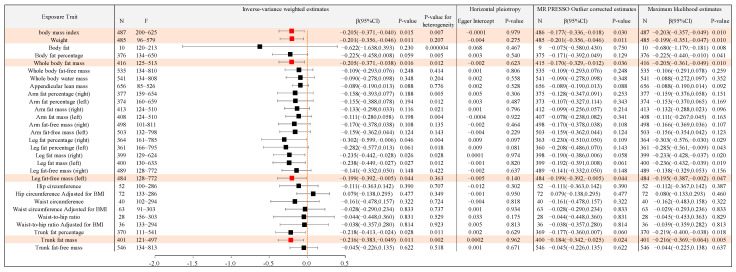
Causal associations between the 29 body anthropometric indexes of obesity and APOE2. (Note: red indicates statistical significance, *p* < 0.05).

**Figure 5:**
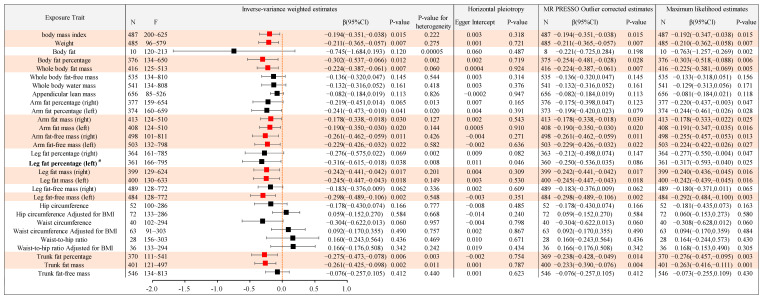
Causal associations between the 29 body anthropometric indexes of obesity and APOE3. (Note: ^#^ indicates horizontal pleiotropy; red indicates statistical significance, *p* < 0.05).

**Figure 6:**
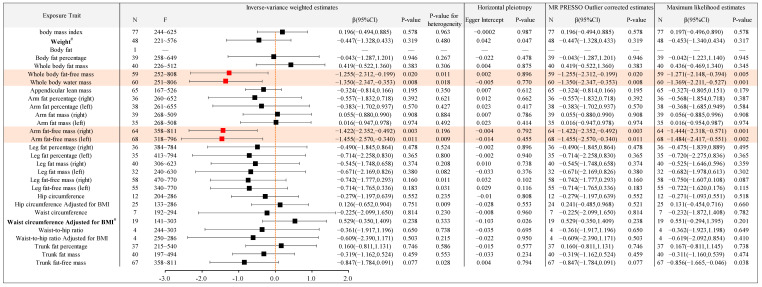
Causal associations between the 29 body anthropometric indexes of obesity and APOE4. (Note: ^#^ indicates horizontal pleiotropy, red indicates statistical significance, *p* < 0.05).

In order to further reveal the causal association between different types of body anthropometric indexes of obesity and the target involved in the top five functional and pathway annotations with the highest frequency, that is, APOE gene polymorphisms, the IVW model was used in the two-sample MR analysis, and the results are shown as follows (Figure 4, Figure 5 and Figure 6).

#### 2.2.1. Causal Association between Different Types of Body Anthropometric Indexes of Obesity and APOE2

Body anthropometric indexes of systemic obesity: Body mass index, weight, whole body fat mass, and leg fat-free mass (left) had negative causal associations with APOE2, with β values of −0.205, −0.201, −0.205, and −0.199, respectively, and all *p*-values were less than 0.05, thereby showing statistical significance. Body fat, body fat percentage, whole body fat-free mass, whole body water mass, appendicular lean mass, arm fat percentage (right), arm fat percentage (left), arm fat mass (right), arm fat mass (left), arm fat-free mass (right), arm fat-free mass (left), leg fat percentage (right), leg fat percentage (left), leg fat mass (right), leg fat mass (left), and leg fat-free mass (right) had no significant causal association with APOE2.

Body anthropometric indexes of central obesity: Trunk fat mass had a negative causal association with APOE2, with a β value of −0.216, and *p*-value of less than 0.05, with statistical significance. Hip circumference, hip circumference adjusted for BMI, waist circumference, waist circumference adjusted for BMI, waist-to-hip ratio, waist-to-hip ratio adjusted for BMI, trunk fat percentage, and trunk fat-free mass all had no significant causal association with APOE2.

#### 2.2.2. Causal Association between Different Types of Body Anthropometric Indexes of Obesity and APOE3

Body anthropometric indexes of systemic obesity: Body mass index, weight, body fat percentage, whole body fat mass, arm fat mass (right), arm fat mass (left), arm fat-free mass (right), arm fat-free mass (left), leg fat mass (right), leg fat mass (left), and leg fat-free mass (left) had negative causal associations with APOE3, with β values of −0.194, −0.211, −0.302, −0.224, −0.178, −0.190, −0.261, −0.229, −0.242, −0.245, and −0.298, respectively, and all *p*-values were less than 0.05, with statistical significance. Body fat, whole body fat-free mass, whole body water mass, appendicular lean mass, arm fat percentage (right), arm fat percentage (left), leg fat percentage (right), and leg fat-free mass (right) had no significant causal association with APOE3.

Body anthropometric indexes of central obesity: Trunk fat percentage and trunk fat mass had negative causal associations with APOE3, with β values of −0.275 and −0.261, respectively, and both *p*-values were of less than 0.05, with statistical significance. Hip circumference, hip circumference adjusted for BMI, waist circumference, waist circumference adjusted for BMI, waist-to-hip ratio, waist-to-hip ratio adjusted for BMI, and trunk fat-free mass had no significant causal association with APOE3.

#### 2.2.3. Causal Association between Different Types of Body Anthropometric Indexes of Obesity and APOE4

Body anthropometric indexes of systemic obesity: Whole body fat-free mass, whole body water mass, arm fat-free mass (right), and arm fat-free mass (left) had negative causal associations with APOE4, with β values of −1.255, −1.350, −1.422, and −1.455, respectively, and all *p*-values were less than 0.05, with statistical significance. Body mass index, body fat, body fat percentage, whole body fat mass, appendicular lean mass, arm fat percentage (right), arm fat percentage (left), arm fat mass (right), arm fat mass (left), leg fat percentage (right), leg fat percentage (left), leg fat mass (right), leg fat mass (left), leg fat-free mass (right), and leg fat-free mass (left) had no significant causal association with APOE4.

Body anthropometric indexes of central obesity: Hip circumference, hip circumference adjusted for BMI, waist circumference, waist-to-hip ratio, waist-to-hip ratio adjusted for BMI, trunk fat percentage, trunk fat mass, and trunk fat-free mass had no significant causal association with APOE4.

Moreover, the above statistically significant results with *p*-values of less than 0.05 (shown in Table 1) were also significant in MR-PRESSO and Maximum likelihood analyses. The MR Egger analysis showed that there was no significant difference between Egger Intercept and 0, with a *p*-value greater than 0.05, indicating no horizontal pleiotropy.

## 3. Discussion

Although many basic and clinical studies have been conducted worldwide on the correlation between obesity and AD in the medical field, the research conclusions and mechanisms are inconsistent. Therefore, to investigate the correlation between obesity and AD, this study screened and obtained 53 common targets of obesity and AD using bioinformatics and conducted functional and pathway annotations. A total of 42 targets were involved in the top five functional and pathway annotations, of which APOE was the target with the highest frequency. There are three alleles of the APOE gene, which are the protective alleles APOE ε2 [19,20] and APOE ε3 [21] and the risk allele APOE ε4 [22]. The distribution of these alleles in the AD population was as follows: ε3 accounted for the majority of APOE gene pool (58%), and ε2 and ε4 accounted for 4% and 38%, respectively [23]. APOE gene is the most common and strong genetic risk factor for AD, indicating that obesity is correlated with AD.

However, the interaction between obesity and APOE to regulate the pathogenesis of AD remains unclear. Therefore, this study adopted functional and pathway annotations for analysis and found that the main biological processes in which the key target APOE was involved included muscle cell proliferation, the regulation of smooth muscle cell proliferation, smooth muscle cell proliferation, and the regulation of lipid localization; the cellular components in which it was involved included plasma lipoprotein particle, lipoprotein particle, protein–lipid complex, high-density lipoprotein particle, and chylomicron; the molecular function in which it was involved was cholesterol transfer activity; and finally, the Reactome pathways with which it was involved included plasma lipoprotein remodeling, chylomicron remodeling, and HDL remodeling. APOE is a class of glycoproteins expressed in a variety of cells, with the highest expression levels in the liver and brain [24]. However, APOE exists in different forms in peripheral circulation and the central nervous system (CNS) due to the blood–brain barrier (BBB) [25]. APOE in peripheral circulation participates in the redistribution and metabolism of triglycerides, cholesterol, cholesterol esters, phospholipids, and other lipids by forming lipoprotein particles, thereby maintaining lipid homeostasis. Although APOE cannot cross the BBB, the APOE in peripheral circulation could regulate brain function either by directly acting on the endothelial cells of the BBB or by indirectly regulating endothelial and neuronal functions through lipid metabolism, atherosclerosis, and peripheral inflammation [26]. The requirement of cholesterol in the brain is relatively constant, which is mainly synthesized in situ by astrocytes (AS). When APOE is released from the cell, ATP-binding cassette transporters (ABCA1 and ABCG1) on the cell surface will transport cholesterol and phospholipids to bind to APOE, forming phospholipid protein particles, which then bind to the receptors on the cell surface to redistribute cholesterol and other phospholipids into neurons. As the APOE activity increases, so does the amount of cholesterol it carries. At the same time, with the increase in cholesterol in the diet, the cholesterol level in the circulatory system increases, which also causes the increase in APOE in the brain [27]. APOE2, APOE3, and APOE4 are three different apolipoproteins. The different amino acid polymorphisms among the alleles change the structure and function of APOE and determine the differential distributions of APOE subtypes in lipoprotein particles. APOE4 mainly exists in triglyceride-rich particles such as chylomicrons and very-low-density lipoproteins (VLDL), whereas APOE2 and APOE3 preferentially exist in high-density lipoproteins (HDL) [28]. Meanwhile, this single amino acid difference also leads to differences in the regulation of lipid binding and receptor binding, oligomerization tendency, and stability of different subtypes [29,30,31,32]. Studies have shown that different APOE subtypes have different abilities in regulating the transportation of cholesterol between nerve cells and astrocytes. APOE2 and APOE3 can effectively complete the transfer of neuronal lipids to astrocytes to protect neurons from the toxic effects of lipid peroxide, while APOE4 is weaker in this ability [33]. Meanwhile, it has been reported that APOE subtypes have different regulatory effects on the cholesterol level in the brain, and cholesterol can regulate the activity of γ-secretase and Aβ production. Compared with APOE4, APOE3 can promote enzyme-mediated Aβ degradation more effectively [34]. Therefore, combined with the functional and pathway annotations obtained from the bioinformatics analysis in this study, it is suggested that obesity may induce the occurrence of AD through the regulation of lipid metabolism by APOE.

Unlike other risk factors for AD, the amino acid sequence of APOE is not disturbed by diseases or other confounding factors, and the APOE phenotype is determined since birth in patients with AD. Thus, comparing the causal associations between obesity and different APOE phenotypes will be helpful in elucidating the relationship between obesity and AD. In the meantime, most existing studies on obesity mainly focused on BMI. Based on the abovementioned knowledge and assumptions, this study adopted Mendelian randomization to explore the potential associations between 29 body anthropometric indexes of obesity and three subtypes of APOE. The results of the analyses showed that there was no consistent trend in fat mass and fat percentage results for the same indicator. The reason may be that when the fat mass is the same but the body weight is different, the body fat percentage will be different. Thus, body fat percentage is a more objective and accurate measure to reflect the fat distribution. At the same time, some inconsistent results were also found in the analysis of fat-free measures: with the increase of whole body fat-free mass, arm fat-free mass (right) and arm fat-free mass (left), APOE4 (the risk gene of AD) decreases significantly, which may lead to the decrease of the risk of AD. However, as the arm fat-free mass (right), arm fat-free mass (left), leg fat-free mass (left) rise, make APOE3 (the protective gene of AD) dropped significantly, moreover, with the increase of leg fat-free mass (left), APOE2 (the protective gene of AD) will also decrease significantly, which may lead to the in-crease of the risk of AD. Analysis of these inconsistent results may be due to the fact that fat-free mass refers to the mass of bone, water, muscle, and other body components excluding fat, of which muscle is a key component, and it has a weak correlation with lipid metabolism, suggesting that fat-free mass may not be a risk factor for obesity-induced AD. Therefore, the following discussion will focus on the associations between body fat percentage and non-fat-free indexes and the different phenotypes of APOE.

For the body anthropometric indexes related to systemic obesity, a 1-Standard Deviation (SD) increase in body mass index (BMI) corresponds to a 20.5% and 19.4% decrease in the β-values for APOE2 and APOE3, respectively, but BMI had no significant causal association with APOE4; a 1-Standard Deviation (SD) increase in Body fat percentage (BFP) corresponds to a 30.2% decrease in the β-values for APOE3, but BFP had no significant causal association with APOE2 and APOE4; arm and leg fat percentages had no significant causal association with APOE2, APOE3, and APOE4. For the body anthropometric indexes related to central obesity, a 1-Standard Deviation (SD) increase in Trunk fat percentage (TFP) corresponds to a 27.5% decrease in the β-values for APOE3, but TFP had no significant causal association with APOE2 and APOE4. Therefore, the current inconsistent conclusions on the correlation between obesity and AD may be mainly due to APOE polymorphism, and different body fat distribution has little influence on it. The expression level of APOE2 in the AD population carrying APOE ε2 allele (4%) is only regulated by BMI, but not by BFP. Thus, the causal association between systemic obesity and APOE2 cannot be accurately determined when only BMI is used as an indicator of systemic obesity. Meanwhile, there was no significant causal association between the central obesity indicator TFP and APOE2, indicating that the risk of AD in patients carrying the APOE ε2 allele was not caused by central obesity, whereas AD population carrying APOE ε3 allele (58%) may be more susceptible to obesity. Both systemic and central obesity indicators have an impact on the disease. With the increase in BMI, BFP, and TFP, the expression level of APOE3, which plays a protective role against AD, decreases, which may lead to an increase in the risk of AD. However, the expression level of APOE4 in the AD population carrying APOE ε4 allele (38%) is not associated with the abovementioned indicators, which suggests that the risk of AD in this population may not be caused by obesity. APOE4 may be an independent risk factor for AD.

In conclusion, due to the controversies in current studies on the correlation between obesity and AD, this study revealed the correlation between obesity and AD at the molecular level using bioinformatics. Obesity induces the development of AD mainly through the regulation of lipid metabolism by APOE. Furthermore, we innovatively revealed the complexity of the association between obesity and AD via Mendelian randomization, which was mainly determined by APOE polymorphism rather than body fat distribution. APOE ε3 carriers, as the largest group in AD population, might be more susceptible to obesity, while the risk of AD in APOE ε2 and APOE ε4 carriers might not be induced by obesity. These findings open up new ideas for the current disputes and controversies to better understand the association between obesity and AD. Meanwhile, attention should be paid to obesity-related indicators of APOE ε3 carriers in the early stage, and they should regulate their body fat as early as possible and reduce the body fat mass to reduce the risk of AD.

## 4. Methods and Materials

### 4.1. Databases and Software

The following databases and software packages were used in this study: the GeneCards database [35] (https://www.genecards.org/, accessed on 5 May 2023), EVenn [36] (http://www.ehbio.com/test/venn/#/, accessed on 5 May 2023), the STRING database [37] (https://string-db.org/cgi/input.pl, accessed on 5 May 2023), IEU OPEN GWAS [38] (https://gwas.mrcieu.ac.uk/, accessed on 5 May 2023), and the R (version 4.1.2) software [39].

### 4.2. Bioinformatics and Mendelian Randomization Analysis of the Mechanism of Obesity-Induced AD

**Figure 7:**
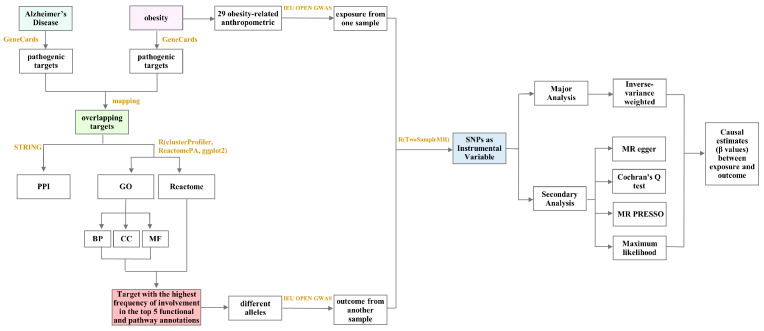
Bioinformatics and Mendelian randomization analysis of the mechanism of obesity-induced AD. (PPI: Protein-Protein Interaction; GO: Gene Ontology; BP: Biological Process; CC: Cellular Com-ponent; MF: Molecular Function; SNPs: Single nucleotide polymorphisms; MR: Mendelian ran-domization).

Figure 7 depicts detailed information.

### 4.3. Bioinformatics Analysis

#### 4.3.1. Screening of Obesity-Related Targets

The GeneCards database was searched using the keywords “obesity” and “overweight”, and the results were exported in an Excel format to filter for obesity-related targets with a relevance score ≥ 7, and were supplemented based on the literature.

#### 4.3.2. Screening of AD-Related Targets

The GeneCards database was screened using the keywords “Alzheimer’s disease”, “Alzheimer”, “Alzheimer disease”, “Alzheimer Dementia”, and “AD”, and the results were exported in an Excel format to filter for AD-related targets with a relevance score ≥ 20, and were supplemented based on the literature.

#### 4.3.3. Protein–Protein Interaction (PPI) Network of Overlapping Targets between Obesity and AD

Obesity- and AD-related targets were imported into EVenn and mapped to each other to obtain the overlapping targets, which were imported into the STRING database to construct PPI network, and the relevant parameters were set as follows: (1) Basic Settings: ① Network type: full network (the edges indicating both functional and physical protein associations); ② meaning of network edges: evidence (line color indicates the type of interaction ev-idence); ③ active interaction sources: Textmining, Experiments, Databases, Co-expression, Neighborhood, Gene Fusion, and Co-occurrence; ④ minimum re-quired interaction score: medium confidence (0.400); ⑤ max number of interactors to show: 1st shell: none/query proteins only, 2nd shell: none. (2) Advanced Settings: ① network display mode: interactive svg; ② network display options: hide disconnected nodes in the network.

In the protein-protein interaction network, the network nodes are proteins. The edges represent the predicted functional associations. In evidence mode, an edge may be drawn with up to 7 differently colored lines, these lines represent the existence of the seven types of evidence used in predicting the associations. Red line indicates the presence of fusion evidence. Green line indicates neighborhood evidence. Blue line in-dicates cooccurrence evidence. Purple line indicates experimental evidence. Yellow line indicates textmining evidence. Light blue line indicates database evidence. Black line indicates coexpression evidence. In confidence mode the thickness of the line indicates the degree of confidence prediction of the interaction.

#### 4.3.4. GO Enrichment and Reactome Pathway Analyses of Overlapping Targets between Obesity and AD

GO enrichment analysis of overlapping targets was performed using the clusterProfiler package in R (version 4.1.2) [40]. Reactome pathway analysis of overlapping targets was performed using the ReactomePA package [41]. The ggplot2 package was used for visualization. The biological processes, location of the reaction in the cell, molecular function involved, and signaling pathways involved were analyzed to elucidate the interrelationship between obesity and AD. The frequency of the relevant targets involved in the top five biological processes, cellular components, molecular functions, and Reactome pathways were also determined to elucidate the key targets that play an important role in obesity-induced AD.

### 4.4. Mendelian Randomization (MR) Analysis

Two-sample Mendelian randomization (2SMR) was performed using large-scale genome-wide association study (GWAS) datasets of diseases and related targets. MR is based on the Mendelian inheritance that alleles segregate randomly during gamete formation, so that that they are not confounded by common factors such as postnatal environment, socioeconomic factors, and behavioral habits, and instead conform to a causal time series, which is more practical and convenient compared to the gold-standard randomized controlled trials (RCTs) for verifying causal associations, and is similar to RCTs in terms of reliability [42,43,44,45].

#### 4.4.1. Data Source

In this study, 29 body anthropometric indexes of obesity were selected as exposure traits to analyze the causal association between them and APOE, the target with the highest frequency of involvement among the top five functional and pathway annotations obtained in Section 2.1.2. Since the APOE gene has significant polymorphism with APOE2, APOE3, and APOE4 as its main alleles [26], the three were selected as the outcome traits. GWAS data for these variables were obtained through the IEU OPEN GWAS platform, and basic information are shown in Table 2 and Table 3. These data were obtained from populations of European origin.

#### 4.4.2. Selection of Instrumental Variables

Single nucleotide polymorphisms (SNPs) are instrumental variables that are the basis for MR studies. In this study, SNPs for the 29 body anthropometric indexes of obesity such as body mass index, weight, and hip circumference were screened separately from the GWAS data shown in Table 2 for MR studies. SNPs were screened based on the following criteria: (1) significant association with risk factors at the genome-wide level (*p* < 5 × 10^−8^) using genome-wide data from the European 1000 Genomes Project as a reference; (2) independence from each other (physical distance within 10,000 kb with linkage disequilibrium r^2^ < 0.001) to avoid possible bias in the analysis due to strong linkage disequilibrium (LD) between SNPs; (3) correlation strength (F-statistic) > 10 with phenotypes to avoid bias from the presence of weak instrumental variables.

#### 4.4.3. Statistical Inference for Causal Effects

The inverse variance weighted (IVW) random effects model was used as the primary analytical method to assess the causal effect between obesity and the target with the highest frequency of involvement in the functional and pathway annotations. The principle of IVW is to weigh the inverse of the variance of each instrumental variable while ensuring that all instrumental variables are valid, and to perform regression without considering the intercept term; the final result is the weighted average of the effect values of all instrumental variables [46]. To assess the robustness of the IVW results, (i) MR-Egger [47] regression was used in order to assess the bias caused by horizontal pleiotropy, in which the intercept indicates the magnitude of horizontal pleiotropy, with an intercept close to 0 indicating minimal pleiotropy; (ii) Cochran’s Q was used to test the difference between individual IVs, and the effect of heterogeneity was considered negligible if the test result was *p* > 0.05; (iii) MR-PRESSO [48] was used to remove abnormal SNPs (outliers) to correct for horizontal pleiotropy to provide more robust estimates; (iv) and IVW causal estimation was complemented using the maximum likelihood model, and the causal effect between exposure and outcome traits was considered robust when the results of the analysis of both the models were statistically significant.

These analyses were performed using the TwoSampleMR package [38] in R (version 4.1.2). The evaluation metrics were β-values and a 95% confidence interval (95% CI). Differences with a two-sided *p* < 0.05 were considered statistically significant.

## Figures and Tables

**Table 1:** Summary table of statistically significant causal effects between anthropometric indexes of obesity and APOE alleles.

IVW-β (95%CI)	APOE2 (4% AD)	APOE3 (58% AD)	APOE4 (38% AD)
body mass index	−0.205(−0.371,−0.040)	−0.194(−0.351,−0.038)	/
Weight	−0.201(−0.356,−0.046)	−0.211(−0.365,−0.057)	/
Body fat percentage	/	−0.302(−0.537,−0.066)	/
Whole body fat mass	−0.205(−0.371,−0.038)	−0.224(−0.387,−0.061)	/
Whole body fat-free mass	/	/	−1.255(−2.312,−0.199)
Whole body water mass	/	/	−1.350(−2.347,−0.353)
Arm fat mass (right)	/	−0.178(−0.338,−0.018)	/
Arm fat mass (left)	/	−0.190(−0.350,−0.030)	/
Arm fat-free mass (right)	/	−0.261(−0.462,−0.059)	−1.422(−2.352,−0.492)
Arm fat-free mass (left)	/	−0.229(−0.426,−0.032)	−1.455(−2.570,−0.340)
Leg fat mass (right)	/	−0.242(−0.441,−0.042)	/
Leg fat mass (left)	/	−0.245(−0.447,−0.043)	/
Leg fat-free mass (left)	−0.199(−0.392,−0.005)	−0.298(−0.489,−0.106)	/
Trunk fat percentage	/	−0.275(−0.473,−0.078)	/
Trunk fat mass	−0.216(−0.383,−0.049)	−0.261(−0.425,−0.098)	/

(Note: / indicates no statistical significance, *p* > 0.05).

**Table 2:** Basic information of exposure traits from the GWAS data.

Exposure Trait	GWAS ID	Sample Size	Number of SNPs	Consortium	PMID	Sex	Year
Body mass index	ieu-b-40	681,275	2,336,260	GIANT	30124842	Males and Females	2018
Weight	ukb-b-11842	461,632	9,851,867	MRC-IEU	—	Males and Females	2018
Body fat	ieu-a-999	100,716	3,228,665	—	26833246	Males and Females	2016
Body fat percentage	ukb-b-8909	454,633	9,851,867	MRC-IEU	—	Males and Females	2018
Whole body fat mass	ukb-b-19393	454,137	9,851,867	MRC-IEU	—	Males and Females	2018
Whole body fat-free mass	ukb-b-13354	454,850	9,851,867	MRC-IEU	—	Males and Females	2018
Whole body water mass	ukb-b-14540	454,888	9,851,867	MRC-IEU	—	Males and Females	2018
Appendicular lean mass	ebi-a-GCST90000025	450,243	18,071,518	—	33097823	Males and Females	2020
Arm fat percentage (right)	ukb-b-12854	454,789	9,851,867	MRC-IEU	—	Males and Females	2018
Arm fat percentage (left)	ukb-b-20188	454,724	9,851,867	MRC-IEU	—	Males and Females	2018
Arm fat mass (right)	ukb-b-6704	454,757	9,851,867	MRC-IEU	—	Males and Females	2018
Arm fat mass (left)	ukb-b-8338	454,684	9,851,867	MRC-IEU	—	Males and Females	2018
Arm fat-free mass (right)	ukb-b-19520	454,753	9,851,867	MRC-IEU	—	Males and Females	2018
Arm fat-free mass (left)	ukb-b-19925	454,672	9,851,867	MRC-IEU	—	Males and Females	2018
Leg fat percentage (right)	ukb-b-20531	454,854	9,851,867	MRC-IEU	—	Males and Females	2018
Leg fat percentage (left)	ukb-b-18377	454,826	9,851,867	MRC-IEU	—	Males and Females	2018
Leg fat mass (right)	ukb-b-18096	454,846	9,851,867	MRC-IEU	—	Males and Females	2018
Leg fat mass (left)	ukb-b-7212	454,823	9,851,867	MRC-IEU	—	Males and Females	2018
Leg fat-free mass (right)	ukb-b-12828	454,835	9,851,867	MRC-IEU	—	Males and Females	2018
Leg fat-free mass (left)	ukb-b-16099	454,805	9,851,867	MRC-IEU	—	Males and Females	2018
Hip circumference	ieu-a-49	213,038	2,559,739	GIANT	25673412	Males and Females	2015
Hip circumference adjusted for BMI	ieu-a-55	211,114	2,540,926	GIANT	25673412	Males and Females	2015
Waist circumference	ieu-a-61	232,101	2,565,408	GIANT	25673412	Males and Females	2015
Waist circumference adjusted for BMI	ieu-a-67	231,353	2,546,074	GIANT	25673412	Males and Females	2015
Waist-to-hip ratio	ieu-a-73	212,244	2,560,782	GIANT	25673412	Males and Females	2015
Waist-to-hip ratio Adjusted for BMI	ieu-a-79	210,082	2,542,432	GIANT	25673412	Males and Females	2015
Trunk fat percentage	ukb-b-16407	454,613	9,851,867	MRC-IEU	—	Males and Females	2018
Trunk fat mass	ukb-b-20044	454,588	9,851,867	MRC-IEU	—	Males and Females	2018
Trunk fat-free mass	ukb-b-17409	454,508	9,851,867	MRC-IEU	—	Males and Females	2018

**Table 3:** Basic information of outcome traits from the GWAS data.

Outcome Trait	GWAS ID	Sample Size	Number of SNPs	Consortium	PMID	Sex	Year
Apolipoprotein E (isoform E2)	prot-a-132	3301	10,534,735	—	29875488	Males and Females	2018
Apolipoprotein E (isoform E3)	prot-a-131	3301	10,534,735	—	29875488	Males and Females	2018
Apo E4	prot-c-2938_55_2	—	501,428	—	28240269	Males and Females	2019

## Data Availability

Publicly available datasets were analyzed in this study. These data can be found here: [GeneCards] at (https://www.genecards.org/), [STRING] at (https://string-db.org/cgi/input.pl), and [IEU OPEN GWAS] at (https://gwas.mrcieu.ac.uk/).

## Abbreviations

2SMR	two-sample Mendelian randomization
AD	Alzheimer’s disease
ALB	Albumin
APOA1	Apolipoprotein A-I
APOB	Apolipoprotein B-100
APOE	Apolipoprotein E
AS	astrocytes
Aβ	amyloid β-protein
BBB	blood–brain barrier
BFP	body fat percentage
BMI	body mass index
BP	biological process
CC	cellular component
CETP	cholesteryl ester transfer protein
CI	confidence interval
CNS	central nervous system
GO	gene ontology
GWAS	genome-wide association study
HDL	high density lipoprotein
IL18	Interleukin 18
IL1B	Interleukin 1 beta
IL6	Interleukin 6
INS	insulin
IVs	instrumental variables
IVW	inverse variance Weighted
LD	linkage disequilibrium
LPL	lipoprotein lipase
MF	molecular function
MR	Mendelian randomization
OR	odds ratio
PON1	serum paraoxonase/arylesterase 1
PPI	protein–protein interaction
PTEN	Phosphatidylinositol 3,4,5-trisphosphate 3-phosphatase and dual-specificity protein phosphatase PTEN
RCT	randomized controlled trial
SD	Standard Deviation
SNPs	single-nucleotide polymorphisms
TFP	trunk fat percentage
TNF	tumor necrosis factors
VLDL	very low-density lipoprotein
WHO	World Health Organization

## References

[1] Alzheimer’s Association (2002). 2022 Alzheimer’s disease facts and figures. Alzheimer’s Dement. J. Alzheimer’s Assoc..

[2] Naderali E.K., Ratcliffe S.H., Dale M.C. (2009). Obesity and Alzheimer’s disease: A link between body weight and cognitive function in old age. Am. J. Alzheimer’s Dis. Other Dement..

[3] Morys F., Potvin O., Zeighami Y., Vogel J., Lamontagne-Caron R., Duchesne S., Dagher A. (2022). Obesity-Associated Neurodegeneration Pattern Mimics Alzheimer’s Disease in an Observational Cohort Study. J. Alzheimer’s Dis..

[4] Profenno L.A., Porsteinsson A.P., Faraone S.V. (2010). Meta-analysis of Alzheimer’s disease risk with obesity, diabetes, and related disorders. Biol. Psychiatry.

[5] Whitmer R.A., Gunderson E.P., Barrett-Connor E., Quesenberry C.P., Yaffe K. (2005). Obesity in middle age and future risk of dementia: A 27-year longitudinal population based study. Br. Med. J..

[6] Beydoun M.A., Beydoun H.A., Wang Y. (2008). Obesity and central obesity as risk factors for incident dementia and its subtypes: A systematic review and meta-analysis. Obes. Rev. Off. J. Int. Assoc. Study Obes..

[7] Berlinger W.G., Potter J.F. (1991). Low Body Mass Index in demented outpatients. J. Am. Geriatr. Soc..

[8] Burns A., Marsh A., Bender D.A. (1989). Dietary intake and clinical, anthropometric and biochemical indices of malnutrition in elderly demented patients and non-demented subjects. Psychol. Med..

[9] Nourhashemi F., Deschamps V., Larrieu S., Letenneur L., Dartigues J.-F., Barberger-Gateau P. (2003). Body mass index and incidence of dementia: The PAQUID study. Neurology.

[10] Qu S., Lu H., Song Y.F. (2021). Multidisciplinary Clinical Consensus on Diagnosis and Treatment of Obesity (2021 Edition). Chin. J. Obes. Metab..

[11] Deng Y.-T., Li Y.-Z., Huang S.-Y., Ou Y.-N., Zhang W., Chen S.-D., Zhang Y.-R., Yang L., Dong Q., Feng J.-F. (2022). Association of life course adiposity with risk of incident dementia: A prospective cohort study of 322,336 participants. Mol. Psychiatry.

[12] Jagust W., Harvey D., Mungas D., Haan M. (2005). Central obesity and the aging brain. Arch. Neurol..

[13] Tang X., Zhao W., Lu M., Zhang X., Zhang P., Xin Z., Sun R., Tian W., Cardoso M.A., Yang J. (2021). Relationship between Central Obesity and the incidence of Cognitive Impairment and Dementia from Cohort Studies Involving 5,060,687 Participants. Neurosci. Biobehav. Rev..

[14] Gustafson D.R., Backman K., Waern M., Ostling S., Guo X., Zandi P., Mielke M.M., Bengtsson C., Skoog I. (2009). Adiposity indicators and dementia over 32 years in Sweden. Neurology.

[15] Fitzpatrick A.L., Kuller L.H., Lopez O.L., Diehr P., O’Meara E.S., Longstreth W.T., Luchsinger J.A. (2009). Midlife and late-life obesity and the risk of dementia: Cardiovascular health study. Arch. Neurol..

[16] Jayedi A., Soltani S., Zargar M.S., Khan T.A., Shab-Bidar S. (2020). Central fatness and risk of all-cause mortality: Systematic review and dose-response meta-analysis of 72 prospective cohort studies. Br. Med. J..

[17] (2000). Obesity: Preventing and managing the global epidemic. Report of a WHO Consultation.

[18] Huang S.-Y., Yang Y.-X., Chen S.-D., Li H.-Q., Zhang X.-Q., Kuo K., Tan L., Feng L., Dong Q., Zhang C. (2021). Investigating causal relationships between exposome and human longevity: A Mendelian randomization analysis. BMC Med..

[19] Shinohara M., Kanekiyo T., Yang L., Linthicum D., Shinohara M., Fu Y., Price L., Frisch-Daiello J.L., Han X., Fryer J.D. (2016). *APOE2* eases cognitive decline during Aging: Clinical and preclinical evaluations. Ann. Neurol..

[20] Zhao L., Gottesdiener A.J., Parmar M., Li M., Kaminsky S.M., Chiuchiolo M.J., Sondhi D., Sullivan P.M., Holtzman D.M., Crystal R.G. (2016). Intracerebral adeno-associated virus gene delivery of apolipoprotein E2 markedly reduces brain amyloid pathology in Alzheimer’s disease mouse models. Neurobiol. Aging.

[21] Sepulveda-Falla D., Sanchez J.S., Almeida M.C., Boassa D., Acosta-Uribe J., Vila-Castelar C., Ramirez-Gomez L., Baena A., Aguillon D., Villalba-Moreno N.D. (2022). Distinct tau neuropathology and cellular profiles of an APOE3 Christchurch homozygote protected against autosomal dominant Alzheimer’s dementia. Acta Neuropathol..

[22] Mahley R.W., Huang Y. (2012). Apolipoprotein e sets the stage: Response to injury triggers neuropathology. Neuron.

[23] Liu C.-C., Kanekiyo T., Xu H., Bu G. (2013). Apolipoprotein E and Alzheimer disease: Risk, mechanisms and therapy. Nat. Rev. Neurol..

[24] Huang Y., Weisgraber K.H., Mucke L., Mahley R.W. (2004). Apolipoprotein E: Diversity of cellular origins, structural and biophysical properties, and effects in Alzheimer’s disease. J. Mol. Neurosci..

[25] Linton M.F., Gish R., Hubl S.T., Bütler E., Esquivel C., I Bry W., Boyles J.K., Wardell M.R., Young S.G. (1991). Phenotypes of apolipoprotein B and apolipoprotein E after liver transplantation. J. Clin. Investig..

[26] Davies N.M., Holmes M.V., Davey Smith G. (2018). Reading Mendelian Randomisation Studies: A Guide, Glossary, and Checklist for Clinicians. BMJ.

[27] Sparks D., Liu H., Gross D.R., Scheff S.W. (1995). Increased density of cortical apolipoprotein E immunoreactive neurons in rabbit brain after dietary administration of cholesterol. Neurosci. Lett..

[28] Weisgraber K.H. (1990). Apolipoprotein E distribution among human plasma lipoproteins: Role of the cysteine-arginine interchange at residue 112. J. Lipid Res..

[29] Ruiz J., Kouiavskaia D., Migliorini M., Robinson S., Saenko E.L., Gorlatova N., Li D., Lawrence D., Hyman B.T., Weisgraber K.H. (2005). The apoE isoform binding properties of the VLDL receptor reveal marked differences from LRP and the LDL receptor. J. Lipid Res..

[30] Hatters D.M., Zhong N., Rutenber E., Weisgraber K.H. (2006). Amino-terminal domain stability mediates apolipoprotein E aggregation into neurotoxic fibrils. J. Mol. Biol..

[31] Yamazaki Y., Painter M.M., Bu G., Kanekiyo T. (2016). Apolipoprotein E as a Therapeutic Target in Alzheimer’s Disease: A Review of Basic Research and Clinical Evidence. CNS Drugs.

[32] Ma Q., Zhao Z., Sagare A.P., Wu Y., Wang M., Owens N.C., Verghese P.B., Herz J., Holtzman D.M., Zlokovic B.V. (2018). Blood-Brain Barrier-Associated Pericytes Internalize and Clear Aggregated amyloid-β42 by LRP1-Dependent Apolipoprotein E Isoform-Specific Mechanism. Mol. Neurodegener..

[33] Liu L., MacKenzie K.R., Putluri N., Maletić-Savatić M., Bellen H.J. (2017). The Glia-Neuron Lactate Shuttle and Elevated ROS Promote Lipid Synthesis in Neurons and Lipid Droplet Accumulation in Glia via APOE/D. Cell Metab..

[34] Osenkowski P., Ye W., Wang R., Wolfe M.S., Selkoe D.J. (2008). Direct and potent regulation of gamma-secretase by its lipid microenvironment. J. Biol. Chem..

[35] Stelzer G., Rosen N., Plaschkes I., Zimmerman S., Twik M., Fishilevich S., Stein T.I., Nudel R., Lieder I., Mazor Y. (2006). The GeneCards Suite: From Gene Data Mining to Disease Genome Sequence Analyses. Curr. Protoc. Bioinform..

[36] Chen T., Zhang H., Liu Y., Liu Y.-X., Huang L. (2021). EVenn: Easy to create repeatable and editable Venn diagrams and Venn networks online. J. Genet. Genom..

[37] Szklarczyk D., Gable A.L., Lyon D., Junge A., Wyder S., Huerta-Cepas J., Simonovic M., Doncheva N.T., Morris J.H., Bork P. (2019). STRING v11: Protein-protein association networks with increased coverage, supporting functional discovery in genome-wide experimental datasets. Nucleic Acids Res..

[38] Hemani G., Zheng J., Elsworth B., Wade K.H., Haberland V., Baird D., Laurin C., Burgess S., Bowden J., Langdon R. (2018). The MR-Base platform supports systematic causal inference across the human phenome. Elife.

[39] R: The R Project for Statistical Computing. https://www.r-project.org/.

[40] The Gene Ontology Consortium (2019). The Gene Ontology Resource: 20 years and still GOing strong. Nucleic Acids Res..

[41] Jassal B., Matthews L., Viteri G., Gong C., Lorente P., Fabregat A., Sidiropoulos K., Cook J., Gillespie M., Haw R. (2020). The reactome pathway knowledgebase. Nucleic Acids Res..

[42] Smith G.D., Hemani G. (2014). Mendelian randomization: Genetic anchors for causal inference in epidemiological studies. Hum. Mol. Genet..

[43] Smith G.D., Ebrahim S. (2003). Mendelian randomization: Can genetic epidemiology contribute to understanding environmental determinants of disease?. Int. J. Epidemiol..

[44] Emdin C.A., Khera A.V., Kathiresan S. (2017). Mendelian Randomization. JAMA.

[45] Yamazaki Y., Zhao N., Caulfield T.R., Liu C.-C., Bu G. (2019). Apolipoprotein E and Alzheimer disease: Pathobiology and targeting strategies. Nat. Clin. Pract. Neurol..

[46] Bowden J., Del Greco M.F., Minelli C., Davey Smith G., Sheehan N., Thompson J. (2017). A framework for the investigation of pleiotropy in two-sample summary data Mendelian randomization. Stat. Med..

[47] Burgess S., Thompson S.G. (2017). Interpreting findings from Mendelian randomization using the MR-Egger method. Eur. J. Epidemiol..

[48] Verbanck M., Chen C.-Y., Neale B., Do R. (2018). Detection of widespread horizontal pleiotropy in causal relationships inferred from Mendelian randomization between complex traits and diseases. Nat. Genet..

